# Multiplex Detection of *Pleurotus ostreatus* Mycoviruses

**DOI:** 10.3390/cimb44110392

**Published:** 2022-11-19

**Authors:** Xiaoyan Zhang, Haijing Hu, Yifan Wang, Junjie Yan, Yu Liu, Jianrui Wang, Xianhao Cheng

**Affiliations:** Shandong Key Laboratory of Edible Mushroom Technology, School of Agriculture, Ludong University, Yantai 264025, China

**Keywords:** *Pleurotus ostreatus*, multiplex PCR, oyster mushroom spherical virus, oyster mushroom isometric virus, *Pleurotus ostreatus* spherical virus, *Pleurotus ostreatus* virus 1

## Abstract

Mycoviruses are viruses that specifically infect and replicate in fungi. Several mycoviruses have been previously reported in *Pleurotus ostreatus*, including the oyster mushroom spherical virus (OMSV), oyster mushroom isometric virus (OMIV), *Pleurotus ostreatus* spherical virus (POSV), and *Pleurotus ostreatus* virus 1 (PoV1). This study was designed to develop a multiplex RT-PCR for simultaneous detection and differentiation of the four *P. ostreatus* mycoviruses. Four pairs of primers were designed from conserved regions based on the reported sequences and the multiplex RT-PCR products were 672 bp for OMSV, 540 bp for OMIV, 310 bp for POSV, and 200 bp for PoV1. The optimal annealing temperature of the multiplex RT-PCR was 62 °C and the detection limits of the plasmids were 100 fg for OMSV and OMIV and 1 pg for POSV and PoV1. This technique was successfully applied for the detection of OMSV, OMIV, and POSV from different *P. ostreatus* strains and the plasmid containing the PoV1 sequence. This methodology can serve as a powerful diagnostic tool for the survey of the incidence and epidemiology of the four *P. ostreatus* mycoviruses, further contributing to the prevention and treatment of mycoviral diseases in *P. ostreatus*.

## 1. Introduction

Mycoviruses, also known as fungal viruses, specifically infect and replicate within fungi. Mycoviruses were discovered relatively late, compared to the viruses of plants, animals, and prokaryotes [1,2,3]. The majority of mycoviruses that have been previously reported contain double-stranded (ds)RNAs genomes, while a small number have single-stranded (ss)RNA or DNA genomes [4]. It is very difficult to identify and characterize new mycoviruses using traditional methods. In recent years, high-throughput sequencing technologies were gradually used for exploration and identification new mycoviruses among fungi [5,6]. The mycovirus was first found and isolated in the diseased mushrooms of *Agaricus bisporus* [7]. With the increasing number of edible mushroom species, several others have been reported to be infected with mycoviruses including *Pleurotus ostreatus*, *Lentinula edodes*, *Flammulina velutipes*, *Cyclocybe aegerita*, *Volvariella volvacea*, *Boletus edulis*, *Armillaria*, *Grifola frondose*, *Auricularia heimuer*, *Leucocybe candicans, Picoa juniperi,* and *Bondarzewia berkeleyi* [3,8,9,10,11,12,13,14,15,16,17,18,19,20,21,22,23]. The majority of known mycoviruses are latent and their infections show no apparent symptoms in their hosts. However, a few mycoviruses have been known to cause damage to their host fungus [24]. In edible mushrooms, mycoviruses often cause severe diseases with symptoms including mycelium degeneration, deformation of fruiting bodies, and reduction in the yield [25,26,27].

*Pleurotus ostreatus* (oyster mushroom) is a widely cultivated edible fungus worldwide, with high nutritional and medicinal values [28]. Several mycoviruses have been previously discovered and identified in *P. ostreatus*, including dsRNA viruses, such as an oyster mushroom isometric virus (OMIV), *Pleurotus ostreatus* spherical virus (POSV), *Pleurotus ostreatus* virus 1 (PoV1), and (the only ssRNA virus) oyster mushroom spherical virus (OMSV) [12,15,29,30,31]. Infection with various mycoviruses differs in the effects on the morphology and physiology of *P. ostreatus*. Recently, studies have shown that an OMSV-Chinese isolate can significantly inhibit mycelial growth, cause fruiting body deformation, and yield loss in *P. ostreatus* [32]. In contrast, *P. ostreatus* infected with PoV1 displayed no distinct morphological or growth phenotypes [29]. The POSV was found and identified in the *P. ostreatus* TD300 strain, which causes strain degeneration [12]. The infection of PoV-ASI2792 influenced the spawn growth and fruiting body development by decreasing the activity of extracellular enzymes such as lignocellulolytic enzymes in *P. ostreatus* [25]. In southern Korea, the OMIV was isolated and characterized from the diseased *P. ostreatus* cultivar Suhan [15]. The diseased *P. ostreatus* cultivar Chunchu coinfected with OMSV and OMIV displayed a delay in mycelial growth, malformations of fruiting bodies, and yield reduction [28].

Mycoviruses have no known natural vectors and are usually horizontally transmitted via hyphal anastomosis and vertically transmitted via sporulation in nature [33]. Studies have shown that Lentinula edodes spherical virus (LeSV) was vertically transmitted by basidiospores in *L. edodes* [34]. A recent transmission study on *A. bisporus* revealed that the mushroom virus X (MVX) can be horizontally transferred via mycelia from an infected strain to five other uninfected strains [35]. Our previous studies have shown that the OMSV can be horizontally transmitted from the OMSV-infected strain to a virus-cured strain [31]. Due to these transmission patterns, it can be a challenge to prevent mycovirus diseases, especially in edible mushrooms. As reported, a few attempts to eliminate mycovirus infections have been described for several edible mushroom species. For example, the methods of growth on a limited nutrient medium containing cAMP and rifamycin, single hyphal tip cultures combined with high-temperature treatment, protoplast regeneration, or mycelial fragmentation were used for curing the OMSV, OMIV, POSV, and PoV-ASI2792 from the virus infected *P. ostreatus* strains [12,28,31,36]. Moreover, ribavirin treatment and mycelial fragmentation were also used for curing Lentinula edodes mycovirus HKB (LeV-HKB) and *L. edodes* partitivirus 1 (LePV1) from *Lentinula edodes* [37]. At present, the most effective control strategy is to detect and eliminate the virus from the virus-infected strain.

PCR is a commonly used technique for the detection and diagnosis of viruses due to its high sensitivity and specificity. Previously, the routine reverse transcription PCR (RT-PCR) technique has been used for the detection of viruses including OMSV, OMIV, PoV1, and POSV. Moreover, serological methods have also been used for the detection of OMSV and OMIV [15,38]. A surface plasmon resonance biosensor chip and a triple antibody sandwich-ELISA system have been successfully developed for the detection of OMSV and OMIV, respectively [15,38]. However, these methods could not distinguish the four *P. ostreatus* mycoviruses in one reaction. Multiplex RT-PCR has become a powerful tool for the simultaneous detection of several different viruses at a time [39,40]. In the present study, we developed a multiplex RT-PCR assay for the simultaneous detection of the four known *P. ostreatus* viruses. This is a simple and effective method to identify single or mixed viral infections in various *P. ostreatus* cultivars. This study represents the first time that the multiplex RT-PCR technique has been used to detect *P. ostreatus* mycoviruses.

## 2. Materials and Methods

### 2.1. Viruses

The *P. ostreatus* strains infected with OMSV, OMIV, POSV, and PoV1 were used for the multiplex RT-PCR detection. The pUC vectors containing genomic sequences of OMSV (Accession No. OL546221), OMIV (Accession No. AY308801.1), POSV (Accession No. GQ505291), and PoV1 (Accession No. NC006961.1 and AY533036.1) were used to investigate the optimal annealing temperature, sensitivity, and specificity of the multiplex PCR detection.

### 2.2. Primer Design

The genomic sequences of OMSV (NC004560.1 and OL546221), OMIV (AY308801.1), POSV (GQ505291.1), and PoV1 (NC006961.1 and AY533036.1) were obtained from the GenBank nucleotide sequence database from the National Center for Biotechnology Information (NCBI, https://www.ncbi.nlm.nih.gov/, accessed on 1 November 2022). Three pairs of specific primers (OMIV-F/R, POSV-F/R, and PoV1-F/R) were designed based on the conserved RNA-dependent RNA polymerase (RdRp) gene sequences of OMIV, POSV, and PoV1, respectively. One pair of primers (OMSV-F/R) were designed in the conserved coat protein (CP) gene of OMSV for the multiplex RT-PCR amplification (Table 1).

### 2.3. RNA Extraction

The *P. ostreatus* strains presented in Table 2 were cultured in solid potato dextrose agar medium. After culturing at 25 °C for one week, 0.1 g mycelia of each strain were collected for RNA extraction by using the RNA Easy Fast Plant Tissue Kit (Tiangen, Beijing, China). The RNA was eluted in 50 μL RNase-free water and stored at −80 °C for future use.

### 2.4. Reverse Transcription

The complementary DNA (cDNA) was synthesized by using a reverse primer and M-MLV reverse transcriptase (Promega, Madison, Wisconsin, USA). The reverse transcription (RT) reaction was performed in a 10 μL RT mixture containing 2 μL total RNA (~1500 ng), 2 μL 5× RT Buffer, 1 μL reverse primers mixture (OMSV-R/OMIV-R/POSV-R/PoV1-R, 10 uM), 0.5 μL dNTP Mixture (2.5 mM each), 0.25 μL RNase Inhibitor (40 U/μL), 0.25 μL MLV Reverse Transcriptase (200 U/μL) and 4 μL RNase Free ddH_2_O. The RT reaction tubes were incubated at 37 °C for 1 h.

### 2.5. PCR Amplification

For uniplex PCR, the 20 μL mixture containing 2 μL cDNA template,10 μL 2× Taq PCR MasterMix II (Tiangen, Beijing, China), 0.5 μL each of forward/reverse primers (10 μM), and 7 μL ddH_2_O. For multiplex PCR, the 20 μL mixture containing 2 μL cDNA template, 10 μL 2× Taq PCR MasterMix II, 4 μL of multiplex primer mixture (containing 0.5 µL each of forward and reverse primer for OMSV, OMIV, POSV, and PoV1, 10 μM), and 4 μL ddH_2_O. A multiplex PCR program was set as follows: pre-denaturation at 95 °C for 5 min, followed by 30 cycles of 3 steps including 95 °C for 30 s, 62 °C for 30 s, followed by 72 °C for 60 s and a final extension at 72 °C for 10 min. After PCR amplification, the products were examined by electrophoresis in 1.5% agarose gel. The healthy *P. ostreatus* sample (virus-cured strain 8129 [31]) acted as a negative control.

### 2.6. Cloning and Sequencing

Each of the mycoviruses infecting *P. ostreatus* was validated by RT-PCR and sequencing. The amplified target fragments of each mycovirus with specific primers were purified and ligated into pMD19-T vector and then transformed into *Escherichia coli* DH5α competent cells. At least three positive recombinant clones were sequenced by Sangon Biotech (Shanghai, China) Co., Ltd.

## 3. Results

### 3.1. Establishment of the Multiplex RT-PCR Assay

**Figure 1:**
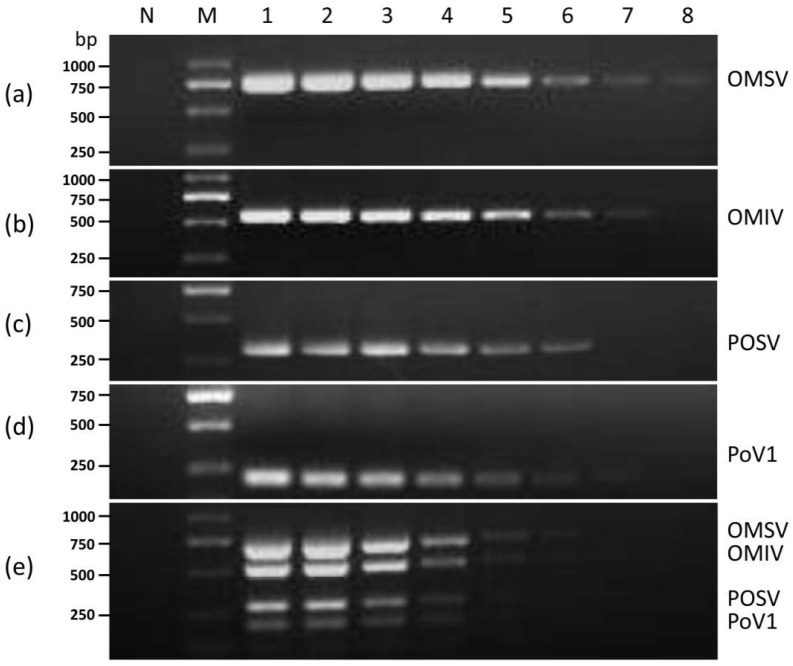
Optimization of the annealing temperature on uniplex RT-PCR detection of (**a**) OMSV (672 bp); (**b**) OMIV (540 bp); (**c**) POSV (310 bp); (**d**) PoV1 (200 bp) and (**e**) multiplex RT-PCR assay. N, negative control, the healthy *P. ostreatus* sample. Lane M: GL DNA Marker2000. Lanes 1–8, 60.0, 61.0, 62.3, 64.0, 66.3, 68.1, 69.3, 70.0 °C.

The primers for OMSV, OMIV, POSV, and PoV1 produced amplicons of 672, 540, 310, and 200 bp, respectively. In order to investigate the effect of the annealing temperature on uniplex and multiplex RT-PCR, a gradient PCR was then performed over a range of 60.0–70.0 °C. Eight annealing temperatures (60.0, 61.0, 62.3, 64.0, 66.3, 68.1, 69.3, and 70.0 °C) were tested for amplification optimization. Gel electrophoresis using ethidium bromide-stained agarose gels were used to separate and visualize the amplified DNA products corresponding to the targeted mycoviruses (Figure 1). When the annealing temperature was 62.3 °C, the amplified bands were bright and clear. However, when the annealing temperatures increased to 64.0 °C, the multiple bands amplified by the multiplex RT-PCR became faint (Figure 1). According to the specificity and efficiency of amplification, the optimal annealing temperature for multiplex RT-PCR was determined to be 62 °C. The PCR program was optimized as follows: initial denaturation at 95 °C for 5 min; 30 cycles of amplification (95 °C for 30 s, annealing at 62 °C for 30 s; elongation at 72 °C for 60 s); and a final extension at 72 °C for 10 min.

### 3.2. Sensitivity of Multiplex RT-PCR

**Figure 2:**
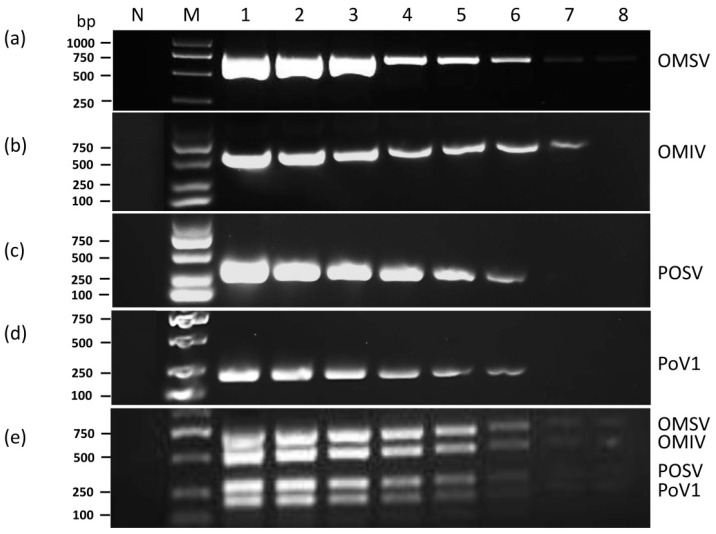
Detection limit based on ten-fold serial plasmid DNA dilutions of uniplex PCR and multiplex PCR detection. Uniplex PCR for (**a**) OMSV; (**b**) OMIV; (**c**) POSV; and (**d**) PoV1; (**e**) multiplex PCR. Lanes 1–8, 10 ng, 1 ng, 10^2^ pg, 10 pg, 1 pg, 10^2^ fg, 10 fg, and 1 fg of plasmids. N, negative control, the healthy *P. ostreatus* sample. Lane M: GL DNA Marker2000. The amplified gene sizes were 672, 540, 310, and 200 bp for OMSV, OMIV, POSV, and PoV1, respectively.

To evaluate the sensitivity of uniplex and multiplex PCR, 10-fold serial dilutions of the purified plasmids pTOMIV, pTPOSV, pTPoV1, and pTOMSV were used as DNA templates in the uniplex and multiplex PCR amplification. The results demonstrated that the detection limits of the DNA quantity for uniplex PCR were 100 fg for OMSV, POSV, PoV1, and 10 fg for OMIV (Figure 2). Using an equal mixture of the four plasmids as templates for multiplex PCR, the detection limits were 100 fg for OMSV and OMIV and 1 pg for POSV and PoV1 (Figure 2).

### 3.3. Specificity of Multiplex RT-PCR

**Figure 3:**
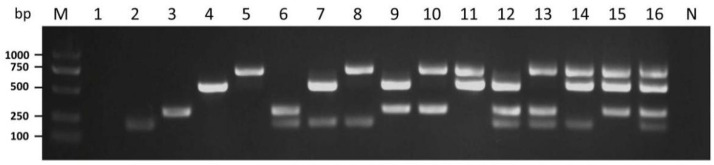
Specificity testing of multiplex PCR with single or mixed templates. Lane 1 shows the empty vector. Lanes 2–16 indicate the plasmids containing genome sequences of the PoV1, POSV, OMIV, OMSV, POSV + PoV1, PoV1+ OMIV, PoV1 + OMSV, POSV + OMIV, POSV + OMSV, OMIV + OMSV, PoV1 + POSV + OMIV; PoV1+ POSV+ OMSV, PoV1 + OMIV + OMSV, POSV + OMIV + OMSV, PoV1 + POSV+ OMIV + OMSV as templates, respectively. N, negative control, the healthy *P. ostreatus* sample. Lane M: GL DNA Marker2000.

To validate the specificity of the multiplex RT-PCR assay, a PCR was conducted by using the mixture of four pairs of primers in one tube, including single or mixed plasmids as templates. It was determined that in the presence of single plasmid, mixtures of primer pairs only amplified the virus corresponding single infection (Figure 3, lines 2–5). Mixed plasmids using dual, triple or quadruple combinations of different templates were also amplified simultaneously using multiplex RT-PCR (Figure 3, lines 6–16). Of all the cases we tested, amplicons were produced by their corresponding mycovirus templates.

### 3.4. Detection of the Four Mycoviruses in Different P. ostreatus Strains

**Figure 4:**
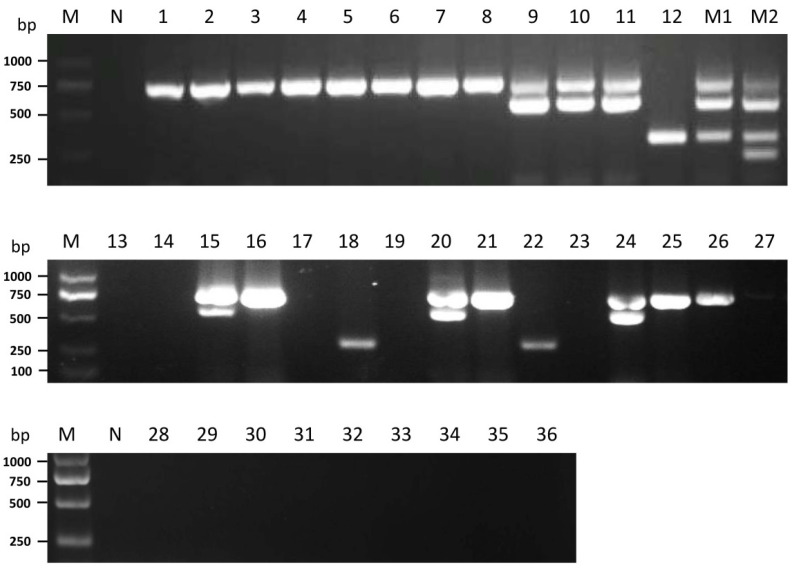
Viruses’ detection of *P. ostreatus* strains using the developed multiplex RT-PCR. Numbers 1–36 represent the 36 strains in Table 2. Lane M1 represents the mixed strains of PG-ZP20 and TD300. Lane M2, combined strains of PG-ZP20 and TD300 with the plasmid pTPoV1. N, negative control, the healthy *P. ostreatus* sample. Lane M: GL DNA Marker2000.

To evaluate specificity for the *P. ostreatus* mycoviruses detection, the established multiplex RT-PCR assay was used to determine the incidence of mycoviruses. The healthy *P. ostreatus* sample (virus-cured strain 8129 [31]) acted as a negative control with no observed virus amplification, as expected. The incidence of both single or mixed infections in different *P. ostreatus* strains produced clear and specific target bands on an agarose gel (Figure 4). Among the 36 *P. ostreatus* strains presented in Table 2, twelve strains were singly infected with OMSV, three strains were singly infected with POSV, and six strains were dually infected with OMSV and OMIV (Figure 4). Although, neither triple nor quadruple viral infection was detected, mixed infections of OMSV, OMIV, and POSV using combinations of the *P. ostreatus* strains PG-ZP20 and TD300 were successfully amplified (Figure 4, line M1). None of the collected 36 strains were infected with PoV1. The above results were confirmed by the four uniplex RT-PCR assays (data not shown). These results demonstrated that the multiplex RT-PCR technique specifically detected the four different mycoviruses in *P. ostreatus*.

## 4. Discussion

Unlike other pathogens such as fungi and bacteria, chemical methods are difficult to employ to control mycoviruses. Therefore, the establishment of a rapid and sensitive detection method is necessary to control the mycovirus disease during the latent stage, or before the ejection of virulent spores. The multiplex RT-PCR assay has the advantage of easy operation, sensitivity, speed, and cost-effectiveness. Thus, this technology has been widely used for the simultaneous identification of several viruses, viral strains, isolates, or genotypes in plant viruses. Previously, this method has been successfully used for simultaneous detection of the two *L. edodes* mycoviruses in China [41]. In the present study, a multiplex RT-PCR assay for simultaneous detection of OMSV, OMIV, POSV, and PoV1 in *P. ostreatus* from one reaction was developed based on the conserved regions of *RdRp* (for OMIV, POSV, and PoV1) and the *CP* (for OMSV) genes. The method presented here demonstrated high sensitivity and specificity for the detection of the four mycoviruses. This methodology can serve as a valuable tool for the diagnosis of the four mycoviruses at the early stages of *P. ostreatus* production. This method can therefore contribute to the prevention and treatment of mycovirus diseases. To the best of our knowledge, this study is the first to report multiplex RT-PCR detection of *P. ostreatus* mycoviruses. Previous reports have demonstrated that some mycoviruses such as Cryphonectria hypovirus 1 (CHV1), Cryphonectria naterciae fusagravirus 1 (CnFGV1), and Cryphonectria nitschkei chrysovirus 1 (CnCV1) can cross the barrier of incompatibility and infect different related species of fungi [42,43,44]. To date, it is not clear whether the four known *P. ostreatus* mycoviruses (OMSV, OMIV, POSV, and PoV1) could infect other closely-related host species. Thus, the multiplex RT-PCR assay can be further used for a comprehensive survey on new host species of the four mycoviruses.

In nature, multiple viral infections are common in plants and this phenomenon also can occur in fungi. An increasing number of fungal species are infected with different mycoviruses. In the plant pathogen *Sclerotium rolfsii*, 21 virus-like sequences have been identified in a hypovirulent strain [45]. In the *L. edodes* mushroom, distinct viral sequences have been previously identified [46]. Co-infection of Lentinula edodes mycovirus HKB (LeV-HKB) with Lentinula edodes partitivirus 1 (LePV1) is prevalent in Chinese *L. edodes* germplasm resources [26,47]. In southern Korea, dual infections of OMSV and OMIV were detected in the *P. ostreatus* cultivars Suhan and Chunchu [15,28]. In the current study, the incidences of the four mycoviruses in different *P. ostreatus* strains were investigated using the multiplex RT-PCR assay. Single infection with OMSV or POSV and dual infection with OMSV and OMIV were detected. Previous research has shown that the incidence rate of OMSV in Beijing suburbs was 32.8% [48]. In the detection of the 36 collected *P. ostreatus* strains, the incidence rate of OMSV was higher (47.2%, 17/36) than OMIV (6/36, 16.7%), POSV (3/36, 8.3%), or PoV1. In order to obtain an accurate prevalence pattern of the four mycoviruses, a larger number of Chinese *P. ostreatus* germplasm resources should be further collected for multiplex RT-PCR detection. In this study, OMIV was found to occur in the form of co-infection with OMSV. Therefore, the possibility that two mycoviruses have synergistic interactions cannot be ruled out. However, whether or not the two mycoviruses have direct interactions requires further study.

Our newly developed multiplex RT-PCR assay offers a simple, quick, and sensitive technique for the diagnosis of the four different mycoviruses infecting *P. ostreatus*. This method can be used as a powerful diagnostic tool for a large-scale survey of the occurrence, distribution, and epidemiology of known *P. ostreatus* mycoviruses. This methodology can contribute to the future prevention and control of mycoviral diseases.

## 5. Conclusions

In this study, we developed a multiplex RT-PCR assay for simultaneous detection and differentiation of the four *P. ostreatus* mycoviruses. This methodology can serve as a powerful diagnostic tool for the survey of the incidence and epidemiology of the four *P. ostreatus* mycoviruses, further contributing to the prevention and treatment of mycoviral diseases in *P. ostreatus*.

## Figures and Tables

**Table 1:** The primers used for the multiplex detection of the four *P. ostreatus* mycoviruses.

Primer Name	Primer Sequence (5′ to 3′)	Target Virus ^a^	Amplicon Size (bp)
OMSV-F	ACCCCCCCAGGATCTCAAGCTTC	OMSV	672
OMSV-R	GAGATGTAGACRTTGAAAGC		
OMIV-F	AACATTGTTGATCACGCTCT	OMIV	540
OMIV-R	GGCTTCAGAATAAAGATTGT		
POSV-F	ATCWCATGGCTATCAACCTA	POSV	310
POSV-R	AGCTGAATTATCGTCACCCA		
PoV1-F	AAACTCGAAGAGTTCCTTTC	PoV1	200
PoV1-R	GCGCGTGGGCCACGTTCGGG		

^a^ OMSV, oyster mushroom spherical virus; OMIV, oyster mushroom isomeric virus; POSV, *Pleurotus ostreatus* spherical virus; PoV1, *Pleurotus ostreatus* virus 1.

**Table 2:** The names, sources, and presence of viruses in this study.

Number	Strain Name	Source	Presence of Virus			
			OMSV	OMIV	POSV	PoV1
1	8129	Yantai	+	−	−	−
2	DF5	Yangzhou	+	−	−	−
3	DF5-2	Liaocheng	+	−	−	−
4	969	Liaocheng	+	−	−	−
5	Kang-2	Liaocheng	+	−	−	−
6	Kang-3	Liaocheng	+	−	−	−
7	P89	Beijing	+	−	−	−
8	P99	Beijing	+	−	−	−
9	PG-0122-1	Yantai	+	+	−	−
10	Heiping	Yantai	+	+	−	−
11	PG-ZP20	Yantai	+	+	−	−
12	TD300	Linyi	−	−	+	−
13	PG-2203	Jinan	−	−	−	−
14	PG-2204	Jinan	−	−	−	−
15	P2108	Dezhou	+	+	−	−
16	LD-0701	Weifang	+	−	−	−
17	LD-0704	Weifang	−	−	−	−
18	LD-0707	Weifang	−	−	+	−
19	LD-0719	Weifang	−	−	−	−
20	LD-1011	Qingdao	+	+	−	−
21	LD-1015	Qingdao	+	−	−	−
22	PGH-1011	Zibo	−	−	+	−
23	PGH-1012	Zibo	−	−	−	−
24	PGH-1014	Zibo	+	+	−	−
25	PGZ-1020	Weihai	+	−	−	−
26	PGZ-1021	Weihai	+	−	−	−
27	PGZ-1022	Weihai	−	−	−	−
28	Huimei	Liaocheng	−	−	−	−
29	Xianfeng-1	Liaocheng	−	−	−	−
30	PG-0324	Yantai	−	−	−	−
31	WPG-1107	Yantai	−	−	−	−
32	8105	Yangzhou	−	−	−	−
33	Luping-0417-5	Yantai	−	−	−	−
34	F803	Linyi	−	−	−	−
35	PG-ZP17	Yantai	−	−	−	−
36	LD-0328	Yantai	−	−	−	−

Note: “+” means infected, and “−” means non-infected.

## Data Availability

The data presented in this study are available within the article.
